# Low-dose green tea intake reduces incidence of atrial fibrillation in a Chinese population

**DOI:** 10.18632/oncotarget.12243

**Published:** 2016-09-24

**Authors:** Dong-Chen Liu, Jian-Jun Yan, You-Nan Wang, Ze-Mu Wang, Zhi-Yong Xie, Yao Ma, Yang Yang, Li Yang, Lian-Sheng Wang

**Affiliations:** ^1^ Department of Cardiology, the First Affiliated Hospital of Nanjing Medical University, Nanjing, Jiangsu, China; ^2^ Department of General Surgery, the First Affiliated Hospital of Nanjing Medical University, Nanjing, Jiangsu, China

**Keywords:** green tea, atrial fibrillation, Chinese, case control, low-dose

## Abstract

The aim of the present study was to assessthe association between green tea intake and incidence of atrial fibrillation (AF) in a Chinese population. A total of 801 (mean age: 62 years; 56% male) subjects were enrolled: 401 AF patients and 400 controls. All subjects completed a questionnaire and the associations between their green tea drinking habits and incidence of AF were assessed using the odds ratio (OR) and binary logistic regression. After multivariate adjustment, green tea intake presented as a protective factor against the incidence of AF (OR: 0.349, 95% CI: 0.253-0.483, *P* < 0.001). The green tea protection showed downward trend with increasing green tea intake (*P* for the trend= 0.001). Low frequency, low concentration, short-term tea consumption was classified as low-dose green tea intake. Green tea intake decreased the incidence of both paroxysmal AF (OR: 0.307, 95% CI: 0.216-0.436, *P* < 0.001) and persistent AF (OR: 0.355, 95% CI: 0.261-0.482, *P* < 0.001) and may be associated with a decreased incidence of AF. This study suggests that low-dose green tea intake strongly protects against AF.

## INTRODUCTION

 Atrial fibrillation (AF) has become a global public health concern. At the end of the last century, the increasing prevalence of AF was mentioned by Braunwald in his Shattuck lecture [1-2]. According to data generated in North America, Britain, and Iceland, the prevalence of AF is approximately 0.5% to 1% in their general populations, respectively [3-5]. AF is the most common cardiac arrhythmia in adults, and is independently related to increased morbidity and mortality [6]. It also causes or exacerbates heart failure and ischemic stroke resulting from thromboembolism [7-10]. In recent years there has been a shift in focus from treatment to prevention and protection in practice guidelines related to AF [11-12].

 The relationship between AF and consumption of certain beverages, such as those containing caffeine and alcohol, has been reported in several recent studies [13-19]. As the most popular beverage consumed around the world, tea intake per capita is approximately 0.12 L/y worldwide [20-21], and green tea was consumed predominantly in Asia [21-22]. Many studies report that green tea plays a protective role in the cardiovascular system [21, 23-26]. Our previous epidemiological studies demonstrate that green tea could protect against the development of coronary heart disease [23]. Moreover, we demonstrated using an *in vitro* experimental model that green tea extract could prevent and treat cardiovascular disease [27]. Habitual intake of tea also has protective effect on coronary heart disease and our meta-analysis revealed that certain teas might decrease the risk of coronary heart disease [21, 25, 28]. As such, we initiated a case-control study to evaluate the hypothesis that green tea intake is inversely related to the incidence of AF.

## RESULTS

### Baseline characteristics

 Table 1 describes the baseline characteristics of the participants in our study. The AF patients and controls displayed significant differences in body mass index (BMI) (*P* < 0.001), hypertension (*P* < 0.001), hyperlipoidemia (*P* = 0.003), and congestive heart failure (*P* < 0.001). The AF patient group had a significantly lower proportion of individuals who drank green tea (38.65%), compared to the control group (60.50%) (*P* < 0.001).

**Table 1:** Baseline characteristics of the participants;

Characteristics	AF (*N* = 401)	Controls (*N* = 400)	Statistical parameter	*P*
Age (years)	63.00±1.24	63.00±1.18	-0.026	0.979
Gender (F/M)	160/241	161/239	0.010	0.920
BMI (kg/m2)	24.32±3.54	23.38±2.80	4.163	<0.001
Hypertension (Y/N)	217/184	122/278	45.747	<0.001
Systolic blood pressure	131.6 ± 24.3	128.6± 21.5	23.712	<0.001
Diastolic blood pressure	78.7±12.1	75.3±11.8	8.403	<0.001
Diabetes (Y/N)	68/333	58/342	0.912	0.339
Hyperlipoidemia (Y/N)	90/311	57/343	8.973	0.003
CAD (Y/N)	105/296	64/336	12.477	<0.001
Smoke (Y/N)	132/269	111/289	2.530	0.112
Drink (Y/N)	144/257	138/262	0.175	0.676
Exercise (Y/N)	195/206	198/202	0.061	0.805
Tea (Y/N) (Y %)	155/246 38.65	242/158 60.5	38.233	<0.001

AF, atrial fibrillation; BMI, body mass index; CAD, coronary heart disease;

### Univariate logistic regression analysis

Univariate analysis of the variables between the patients and controls (Table 2) identified a significant association between AF and green tea consumption (OR: 0.411, 95% CI: 0.310-0.546, *P* < 0.001). Moreover, patients with hypertension (OR: 2.687, 95% CI: 2.012-3.590, *P* < 0.001), hyperlipoidemia (OR: 1.741, 95% CI: 1.208-2.510, *P* = 0.003), or congestive heart failure (OR: 1.862, 95% CI: 1.315-2.637, *P* < 0.001) were more likely to suffer from AF. BMI (OR: 2.755, 95% CI: 1.966-3.861, *P* < 0.001) was an additional risk factor for AF.

**Table 2:** Univariate logistic regression for influencing factors of atrial fibrillation;

Characteristic	All subjects			
		OR	95%CI	*P*
Age		1.200	0.903, 1.594	0.209
Sex (male = 1.female = 0)		1.015	0.765, 1.346	0.920
BMI		2.755	1.966, 3.861	<0.001
Hypertension (yes = 1.no = 0)		2.687	2.012, 3.590	<0.001
Diabetes (yes = 1.no = 0)		1.204	0.822, 1.763	0.340
Hyperlipidemia (yes = 1.no = 0)		1.741	1.208, 2.510	0.003
CAD (yes = 1.no = 0)		1.862	1.315, 2.637	<0.001
Tea(yes = 1.no = 0)		0.411	0.310, 0.546	<0.001
Smoking status (yes = 1.no = 0)		1.278	0.944, 1.728	0.112
Drinking status (yes = 1.no = 0)		1.064	0.796, 1.422	0.676
Exercise (yes = 1.no = 0)		0.966	0.732, 1.274	0.805

1. AF, atrial fibrillation; BMI, body mass index; CAD, coronary heart disease;

### Multivariate logistic regression analysis

We performed a multivariate logistic regression to refine the variables that impact AF. Table 3 shows the results of adjusted OR, respective confidence intervals at 95%, and p value under the multivariate model, including variables of green tea intake, age, sex, BMI, exercise, smoking status, drinking status hypertension, hyperlipoidemia, congestive heart failure and diabetes. We found hypertension (OR: 2.330, 95% CI: 1.679-3.233, *P*< 0.001) and BMI (OR: 1.062, 95% CI: 1.008-1.118, *P* = 0.023) remained associated with an increased incidence of AF. Green tea intake (OR: 0.355, 95% CI: 0.261-0.482, *P* < 0.001) remained an independent protective factor against AF in the whole population.

**Table 3:** Multivariate logistic regression for factors influencing AF;

Characteristics	OR	95%CI	*P*
Age	0.996	0.983-1.010	0.594
Sex(male=1.female=0)	1.101	0.757-1.601	0.615
BMI	1.062	1.008-1.118	0.023
Hypertension(yes=1.no=0)	2.330	1.679-3.233	<0.001
CAD(yes=1.no=0)	1.466	0.992-2.164	0.055
tea(yes=1.no=0)	0.349	0.253-0.483	<0.001
Hyperlipidemia(yes=1.no=0)	1.162	0.765-1.765	0.481
Diabetes(yes=1.no=0)	0.891	0.584-1.360	0.593
exerise(yes=1.no=0)	1.172	0.974-1.410	0.093
Smoking status (yes=1.no=0)	1.235	0.978-1.559	0.076
Drinking status (yes=1.no=0)	1.008	0.784-1.296	0.949

1. AF, atrial fibrillation; BMI, body mass index; CAD coronary artery disease;

2. Multivariate logistic regression for age, gender, body mass index, smoking status, alcohol use, physical activity, hypertension, hyperlipidemia, diabetes mellitus, and CAD in the analysis.

### Relationship between green tea and incidences of AF

 To further confirm the relationship between AF and green tea, we reviewed additional parameters in the questionnaire. We documented the characteristics of the participants' green tea intake, including frequency, concentration, and duration (length of time consistently drinking tea) as described in Table 4. To the best of our knowledge, green tea intake played a protective role against the incidence of AF with a crude OR of 0.411 (*P* < 0.001) and an adjusted OR of 0.385 (*P* < 0.001) after adjustment for age, gender, BMI, smoking status, alcohol use, physical activity, hypertension, hyperlipidemia, diabetes mellitus, and coronary artery disease (CAD). Participants in the low frequency group (adjusted OR: 0.398, 95% CI: 0.223-0.712) had a decreased incidence of AF, compared to those in the high frequency group (adjusted OR: 0.594, 95% CI: 0.285-1.239). Moreover, a dose-response relation was clearly observed (P for the linear trend < 0.001). A similar dose-response relation was found with duration (P for the trend < 0.001). In the concentration groups, no clear dose-response relation was observed (adjusted OR: 1.140, 95% CI: 0.671-1.937), even though the low concentration group showed a protective effect (adjusted OR: 0.223, 95%CI: 0.156-0.317, *P* < 0.001), which suggested low concentration green tea intake decreases the incidence of AF.

**Table 4:** Relationship between green tea intake and risk of AF;

Characteristics	Controls (*N*=400)	Atrial fibrillation (*N* = 401)	Crude OR	*P*	Adjusted OR	*P*
Tea(Y/N)						
0	158	246	1			
1	242	155	0.411 (0.310-0.546)	<0.001	0.385 (0.286-0.519)	<0.001
Frequency						
None	158	246	1		1	
Low	37	23	0.399 (0.229-0.697)	0.001	0.398 (0.223-0.712)	0.002
Moderate	188	115	0.393 (0.289-0.697)	<0.001	0.364 (0.264-0.502)	<0.001
Long	17	17	0.642 (0.319-1.295)	0.216	0.594 (0.285-1.239)	0.165
P for the trend				<0.001		<0.001
Concentration						
None	158	246	1		1	
Low	193	70	0.233 (0.166-0.337)	<0.001	0.223 (0.156-0.317)	<0.001
Moderate	23	34	0.949 (0.539-1.672)	0.857	0.844 (0.469-1.159)	0.572
High	26	51	1.260 (0.754-2.104)	0.377	1.140 (0.671-1.937)	0.628
P for the trend				0.320		0.153
Duration						
Never	158	246	1		1	
Short	61	24	0.253 (0.151-0.422)	<0.001	0.271 (0.160-0.462)	<0.001
Moderate	122	63	0.333 (1.231-0.477)	<0.001	0.319 (0.218-0.465)	<0.001
Long	59	68	0.743 (0.495-1.106)	0.148	0.624 (0.408-0.953)	0.029
P for the trend				<0.001		<0.001

AF, atrial fibrillation; OR, odds ratio; CI, confidence interval;

1. In frequency, group was categorized as low (<1cup/day) moderate (1cup/day) and high (>1cup/day).

2. In concentration, group was categorized as low (tea leaves were <25% of the volume of the cup), moderate (tea leaves were 25–50% of the volume of the cup) and high (tea leaves were >50% of the volume of the cup).

3. In duration, group was categorized as short (1-15Y) moderate (16-30Y) and long (>30Y).

4. Adjustment for age, gender, body mass index, smoking status, alcohol use, physical activity, hypertension, hyperlipidemia, diabetes mellitus, and CAD in the analysis.

In order to further explore the relationship between dose of green tea intake and AF, the green tea drinkers were scored and grouped in terms of duration, frequency and concentration of green tea intake. The frequency of green tea intake was rated on a 3-point scale ranging from low = “1” to high = “3”, the same to concentration and duration accordingly. According to the final score, the green tea drinker was divided into 7 groups ranging from “3” to “9”. The green tea protection showed downward trend with increasing green tea intake (*P* for the trend < 0.001) ranging from “3” to “9”.

**Table 5:** Relations between doses of Green tea intake and AF;

Groups	Controls (*N* = 242)	Atrial fibrillation (*N* = 155)	OR 95% CI	*P*
0	158	246	1	
3	22	3	0.088 (0.026-0.297)	<0.001
4	45	18	0.257 (0.144-0.460)	<0.001
5	92	34	0.237 (0.153-0.369)	<0.001
6	47	39	0.533 (0.333-0.852)	<0.001
7	16	29	1.164 (0.612-2.213)	0.643
8	17	27	1.020 (0.538-1.932)	0.951
9	3	5	1.070 (0.252-4.542)	0.926
P for the trend				<0.001

1. AF, atrial fibrillation; OR, odds ratio; CI, confidence interval;

2. The frequency of green tea intake was rated on a 3-point scale ranging from low = “1” to high = “3”, the same to concentration and duration accordingly.

3. According to the final score, the green tea drinker was divided into 7 groups ranging from “3” to “9.

To explore differences in consumption of green tea among patients with different types of AF, we evaluated the baseline characteristics of our cohort, presented in Table 6. Significant differences in age (*P* < 0.001), BMI (*P* < 0.001), hypertension (*P* = 0.001), hyperlipoidemia (*P* = 0.003), and congestive heart failure (*P* < 0.001) were observed among the 3 AF types. Specifically, permanent AF patients were more likely to be older with lower BMI.

**Table 6:** Characteristics for different atrial fibrillation groups;

Characteristics	Paroxysmal AF (*N* = 240)	Persistent AF (*N* = 72)	Permanent AF (*N* = 89)	Controls (*N* = 400)	Statistical parameter	*P*
Tea (Y/N)	81/159	24/48	50/39	242/158	52.292	<0.001
Age (years)	61.00±1.26	61.00±1.19	67.00±1.13	63.00±1.18	6.098	<0.001
Gender (F/M)	99/141	31/41	30/59	161/239	1.912	0.591
BMI (kg/m2)	24.34±3.69	24.93±2.96	23.76±3.52	23.38±2.80	7.609	<0.001
Hypertension (Y/N)	125/115	40/32	52/37	122/278	46.892	<0.001
Diabetes(Y/N)	37/203	12/60	19/70	58/342	2.641	0.450
Hyperlipoidemia (Y/N)	63/177	16/56	11/78	57/343	17.336	0.001
CAD (Y/N)	63/177	11/61	31/58	64/336	21.620	<0.001
Smoke (Y/N)	81/159	18/54	33/56	111/289	5.474	0.140
Drink (Y/N)	88/152	27/45	29/60	138/262	0.746	0.862
Exercise (Y/N)	115/125	38/34	42/47	198/202	0.679	0.878

AF, atrial fibrillation; BMI, body mass index; CAD, coronary heart disease;

Among the 240 patients with paroxysmal AF, 81 drank green tea. The frequency, concentration, and duration of green tea consumption and multivariable-adjusted associations between green tea intake and paroxysmal AF are described in Table 7. Interestingly, we found green tea intake is a protective factor against paroxysmal AF with a crude OR of 0.333 (95% CI, 0.238-0.465, *P* < 0.001) and an adjusted OR of 0.307 (95% CI, 0.216-0.436, *P* < 0.001). Notably, significant associations were observed between the frequency, concentration, and duration of green tea intake and paroxysmal AF (adjusted *P* for trend < 0.001 for frequency, *P* = 0.005 for concentration, *P* < 0.001 for duration).

**Table 7:** Relationship between green tea intake and risk of paroxysmal AF;

Groups	Controls (*N* = 400)	Paroxysmal AF (*N* = 240)	Crude OR	*P*	Adjusted OR	*P*
Tea (Y/N)						
0	158	159	1			
1	242	81	0.333 (0.238-0.465)	<0.001	0.307 (0.216-0.436)	<0.001
Frequency						
None	158	159	1		1	
Low	37	11	0.295 (0.146-0.600)	0.001	0.301 (0.145-0.625)	0.001
Moderate	188	62	0.328 (0.228-0.471)	<0.001	0.299 (0.205-0.437)	<0.001
High	17	8	0.468 (0.196-1.115)	0.086	0.410 (0.165-1.018)	0.055
P for the trend				<0.001		<0.001
Concentration						
None	158	159	1		1	
Low	193	37	0.191 (0.126-0.288)	<0.001	0.179 (0.116-0.275)	<0.001
Moderate	23	21	0.907 (0.483-1.706)	0.763	0.851 (0.442-1.638)	0.629
High	26	23	0.897 (0.481-1.606)	0.675	0.743 (0.396-1.393)	0.354
P for the trend				0.016		0.005
Duration						
Never	158	159	1		1	
Short	61	8	0.130 (0.060-0.281)	<0.001	0.139 (0.063-0.305)	<0.001
Moderate	122	35	0.285 (0.184-0.441)	<0.001	0.271 (0.173-0.426)	<0.001
Long	59	38	0.640 (0.403-1.017)	0.059	0.532 (0.326-0.867)	0.011
P for the trend				<0.001		<0.001

AF, atrial fibrillation; OR, odds ratio; CI, confidence interval;

1. In frequency, group was categorized as low (<1cup/day) moderate (1cup/day) and high (>1cup/day).

2. In concentration, group was categorized as low (tea leaves were <25% of the volume of the cup), moderate (tea leaves were 25–50% of the volume of the cup) and high (tea leaves were >50% of the volume of the cup).

3. In duration, group was categorized as short (1-15Y) moderate (16-30Y) and long (>30Y).

4. Adjustment for age, gender, body mass index, smoking status, alcohol use, physical activity, hypertension, hyperlipidemia, diabetes mellitus, and CAD in the analysis.

In regard to persistent AF, 24 green tea drinkers and 48 non-tea drinkers are described in Table 8. Using exploratory analyses, we observed that green tea was associated with a protective effect for persistent AF patients (adjusted OR: 0.304, 95% CI: 0.175-0.527, *P* < 0.001). Furthermore, linear trend associations were revealed for frequency (adjusted *P* for trend < 0.001), concentration (adjusted P for trend = 0.001) and duration (adjusted P for trend = 0.002) with persistent AF.

Among different frequencies, concentrations, and durations of tea intake, the ORs were all less than 1, which suggests that the probabilities of paroxysmal AF and persistent AF in the tea group were less than in the non-tea group. There was no statistical link between permanent AF and tea drinking.

**Table 8:** Relationship between green tea intake and risk of persistent AF;

Characteristics	Controls (*N* = 400)	Persistent AF(*N* = 72)	Crude OR	*P*	Adjusted OR	*P*
Tea(Y/N)						
0	158	48	1		1	
1	242	24	0.326 (0.192-0.554)	<0.001	0.304 (0.175-0.527)	<0.001
Frequency						
None	158	48	1		1	
Low	37	7	0.623 (0.261-1.486)	0.286	0.715 (0.289-1.768)	0.468
Moderate	188	15	0.263 (0.142-0.487)	<0.001	0.235 (0.124-0.447)	<0.001
High	17	2	0.387 (0.086-1.736)	0.215	0.318 (0.068-1.495)	0.147
P for the trend				<0.001		<0.001
Concentration						
None	158	48	1		1	
Low	193	17	0.290 (0.160-0.524)	<0.001	0.291 (0.158-0.535)	<0.001
Moderate	23	5	0.716 (0.258-1.984)	0.520	0.506 (0.174-1.475)	0.212
High	26	2	0.253 (0.058-1.106)	0.068	0.190 (0.042-0.869)	0.032
P for the trend				0.002		0.001
Duration						
Never	158	48	1		1	
Short	61	7	0.378 (0.162-0.880)	0.024	0.398 (0.166-0.954)	0.039
Moderate	122	13	0.351 (0.182-0.676)	0.002	0.333 (0.168-0.657)	0.002
Long	59	4	0.223 (0.077-0.646)	0.006	0.175 (0.058-0.524)	0.002
P for the trend				<0.001		<0.001

AF, atrial fibrillation; OR, odds ratio; CI, confidence interval;

1. In frequency, group was categorized as low (<1cup/day) moderate (1cup/day) and high (>1cup/day).

2. In concentration, group was categorized as low (tea leaves were <25% of the volume of the cup), moderate (tea leaves were 25–50% of the volume of the cup) and high (tea leaves were >50% of the volume of the cup).

3. In duration, group was categorized as short (1-15Y) moderate (16-30Y) and long (>30Y).

4. Adjustment for age, gender, body mass index, smoking status, alcohol use, physical activity, hypertension, hyperlipidemia, diabetes mellitus, and CAD in the analysis.

## DISCUSSION

### Principal study

Herein, we present a case-control study evaluating the associations between green tea intake and the incidence of AF in a Chinese population. We observed a statistically significant relationship between green tea intake and reduced incidence of AF following multivariable adjustment.

The results of our study suggest that green tea intake might decrease the incidence of AF in accordance with a previous report [29]. Coronary heart disease is often considered to be an independent factor affecting the atrial fibrillation. Wang ZM *et al* reported a tentative association of green tea consumption with a reduced risk of CAD in a meta-analysis [21]. Pang *et al* provided evidence in the meta-analyses, included 9 studies and 259,267 individuals, that green tea intake is associated with favorable outcomes with respect to risk of cardiovascular diseases, myocardial infarction and stroke [30]. Hypertension is commonly thought to increase the incidence of atrial fibrillation. Greyling *et al* reported a reduction in blood pressure after tea intake in the meta-analysis by summaring evidence from 11 randomized controlled intervention studies including 378 subjects, [31]. It has been well established that atrial remodeling is a mechanism for promoting AF [32]. The processes of oxidative stress and inflammation could play important roles in increased fibrosis and inflammation and/or alteration of electrophysiology which could [7, 8]. In fact, these associations have been validated in numerous clinical and experimental studies [33-39]. Patients with AF exhibit high NF-kappaB activity, high concentrations of TNF-alpha and IL-6, severe lymphomonocyte infiltration, and fibrosis [40]. Wu *et al* reported increased C-reactive protein (CRP), interleukin-6 (IL-6), and tumor necrosis factor-alpha were significantly associated with AF risk [41-42]. Catechins constitute 80-90% of total flavonoids, the main ingredient of green tea, with epigallocatechin gallate (EGCG) being the most abundant (48-55%) [24, 43]. EGCG has been demonstrated to play an important anti-oxidative, anti-proliferative, and anti-inflammatory role in cardiovascular diseases [44]. EGCG has also been reported to have anti-NF-κB transactivation activity in chronic inflammation [45], preventing PCB-126-induced endothelial cell inflammation *via* epigenetic modifications of NF-κB target genes in human endothelial cells [46]. Ras *et al* by reported a meta-analysis moderate consumption of tea substantially enhances endothelial-dependent vasodilation [47].Our previous investigation reported that EGCG significantly suppresses the TNF-alpha-induced MCP-1 protein and mRNA expression, thereby inhibiting inflammation and anti-oxidative stress [27]. Zhu *et al* reported that EGCG protected against atrial electrical and structural remodeling in a rabbit RAP model by attenuating inflammation and oxidative stress [48]. Therefore, EGCG could be the most important contributor to the beneficial effect of green tea on AF incidence.

In the present study, we analyzed the tea drinking habits of a Chinese population. Our results demonstrate that frequency and duration of drinking green tea are equivocally associated with a decreased incidence of AF. The results also suggest that excessive green tea intake weakens the protective effect. We speculated another component of green tea might reduce the effect of EGCG. Green tea contains large quantities of caffeine which is not directly associated with decreased incidence of AF [13, 49- 50]. High frequency-high concentration, or long-term green tea consumption may lead to high cumulative doses of caffeine which might contribute to an incidence of AF [51]. In the long duration and high frequency groups, a negative correlation, corresponding to the increase of OR value, indicates that the protective effect is weakened. The high tea concentration group has an OR greater than 1, indicating that the effect is not protective but harmful. These results suggest that low-dose green tea induces a strong protective effect and increasing tea consumption weakens this protection.

Subsequently, we divided participants according to different AF types. We found green tea intake is linearly correlated with decreased incidence of both paroxysmal AF and persistent AF, with an adjustment OR less than 1. However, green tea intake is not associated with permanent AF. The paroxysmal and the persistent AF were in the early and middle stages of oxidative stress, inflammation, fibrosis and alteration of electrophysiology [52]. As such, EGCG may result in an anti-inflammatory, anti-oxidation, and anti-fibrosis effect. Participants with permanent AF displayed severe electrophysiological and organic changes [53], which may preclude any benefit of green tea. We found that increased frequency, concentration, and duration of green tea intake had a negative correlation with protection from paroxysmal AF, which is consistent with the overall results, but a positive correlation with protection of persistent AF. It has been reported that caffeine does not affect prevailing cardiac rhythm and rate in healthy adults [54]. Moreover, elevated levels of sympathetic activity have been shown to be detrimental [51, 55]. Thus, while caffeine intake might be associated with increased incidence of AF, data remains inconclusive at this point in time [49]. Persistent AF risk for patients decreased with the increasing frequency, duration and concentration of green tea consumed. When AF becomes persistent, it is changed and associated with atrial enlargement and structural changes [56-57], and increasing fibrosis [58], which could enhance the protective role of EGCG for some unknown reason.

Our study supports low-dose green tea intake as a means of protecting against persistent AF and paroxysmal AF. The underlying mechanism by which green tea affects its function in reducing the incidence of AF will require further experimental studies.

### Advantages and limitations

To the best of our knowledge, this work represents the first study on the association between green tea intake and the incidence of AF. We interpreted the relation between green tea and different types of AF with a consistent conclusion that green tea, especially when consumed in small amounts, provides a protective effect. These observations may aid in the prevention of the occurrence of AF or protection after its occurrence in clinical .

There are, however, several potential limitations in this research. First, our research was a cross-sectional, single-center observational study with a relatively small sample size. Thus, these results need to be validated in a large-scale, multi-center prospective study. Second, our study was a retrospective case-control study and thus susceptible to various biases. Green tea intake was assessed by the number of cups consumed daily or weekly, however, the size of cups was not uniform. Moreover, memory bias is inevitable and uncontrolled confounders cannot be ruled out as a potential effect on the relationship between green tea intake and incidence of AF; such confounders could include coffee consumption, drug and herbal medicine intake, and psychological factors. Third, screening electrocardiograms were performed only once, and it is possible that asymptomatic AF may have gone undetected in the control group. Fourth, we have not yet confirmed the mechanism by which green tea exerts its protective role. Finally, our study was based entirely on a Chinese population; it is uncertain whether the results can be extrapolated to other populations.

In conclusion, green tea drinking appears to be independently associated with lower incidence of AF in this large cohort of Chinese patients. Our results suggest that low-dose green tea intake could provide a protective effect; however, additional multi-center large-scale prospective cohort studies are required to validate our results. Ultimately, the association between routine intake of green tea and reduced incidence of AF is promising and warrants further research.

## MATERIALS AND METHODS

### Participants

Eight-hundred one (*n* = 801) participants (480 males and 321 females) between the ages of 17 and 89 were recruited from First Affiliated Hospital of Nanjing Medical University from September 2014 to October 2015. This study was approved by the Ethics Committee of the First Affiliated Hospital of Nanjing Medical University, Jiangsu Province, China, and written informed consent was received from each patient. The study of green tea intake and AF was in accordance with approved institutional guidelines. Eligible participants diagnosed with AF were randomly recruited as a case group. We collected information *via* a questionnaire, which was distributed to 650 patients and completed by 469 individuals. To minimize bias, we ruled out 30 patients without reliably confirmed disease and the possibility of secondary AF, including patients with history of valvular disease (*n* = 6), cardiomyopathy (*n* = 9), congenital heart disease (*n* = 4), and hyperthyroidism (*n* = 8). Finally, the case group consisted of 401 participants (241 males and 160 females), and 400 (239 males and 161 females) individuals without history of AF as the control group.

### Ascertainment of incidence of AF

AF patients enrolled in the investigation presented with a history of paroxysmal AF as documented by electrocardiography and AF details collected through review of medical records with informed consent. Subjects were divided into the following 4 groups: 1) no AF; 2) paroxysmal AF; 3) persistent AF; and 4) permanent AF.

If the arrhythmia terminated spontaneously and within 7 days, AF was designated as paroxysmal; and when sustained beyond 7 days or terminated by pharmacological or electrical cardioversion, AF was deemed persistent. Persistent AF also includes cases of longstanding AF, usually leading to permanent AF, in which cardioversion has failed or has been foregone [59-60].

### Assessment of green tea intake and other covariates

Information on green tea intake and other baseline covariates were collected *via*on the 801 participants. Participants were divided into 2 groups based on their response to the question ‘Do you drink green tea?’ (Y/N). The yes-group was followed up with several questions which included: (1) How often do you drink green tea each week? (2) How long have you been drinking tea? (3) What concentration of green tea do you usually consume? Based on the answers provided we created 4 categories of frequency: none, < 1, 1, and > 1 cup/week, and 4 categories of duration: none, < 15, 16-30, > 30years. The concentration was divided into low (green tea leaves were < 25% of the wet volume of the cup), moderate (green tea leaves were 25-50% of the wet volume of the cup) and high (green tea leaves were > 50% of the wet volume of the cup) [23]. The frequency of green tea intake was rated on a 3-point scale ranging from low = “1” to high = “3”, the same to concentration and duration accordingly. According to the final score, the green tea drinker was divided into 7 groups ranging from “3” to “9”. Accordingly, we defined the group of 3 points as low-dose green tea intake of group.

The clinical characteristics of participants were collected, including age (in years), gender (male, female), BMI (calculated from height and weight; in kg/m^2^), physical activity (Y/N), smoking status (Y/N), alcohol consumption (Y/N), physician-diagnosed hypertension (Y/N), physician-diagnosed diabetes mellitus (Y/N), physician-diagnosed hyperlipoidemia (Y/N), and physician-diagnosed coronary heart disease (Y/N).

### Statistical analyses

Statistical analysis was conducted using Statistics Package for Social Sciences (ver. 16.0; SPSS Incorporated, Chicago, IL, USA). We analyzed the basic characteristics of the overall data. Continuous variables of age and BMI were normally distributed and are presented as mean ± SD. The comparisons were analyzed using variance analysis. Sex, smoking status, drinking status, physical activity, CHD status, diabetes status, hypertension status, and hyperlipoidemia status were treated as categorical variables and compared among the groups of patients using chi-squared analyses. We used univariate and multiple logistic regression analyses to access and exclude various factors influencing the interference. To facilitate analysis, we regarded non-tea drinkers as the reference group, and categorized all green tea intake variables, except the response to ‘do you drink green tea (Y/N)?’, into several levels [61]. We calculated odds ratios (ORs) and 95% confidence intervals (CIs) to express the association, and each quantitative categorization of green tea intake was subjected to a linear trend test. Subsequently, AF patients were grouped according to their classification (paroxysmal, persistent, or permanent) of AF, as previously described. We calculated ORs and 95% CIs and conducted a linear trend test to reveal the association between green tea intake and different types of AF.

## FINANCIAL SUPPORT

This work was supported by grants from the National Natural Science Foundation of China (No 81270255 & 81570363) and a Project Funded by the Priority Academic Program Development of Jiangsu Higher Education Institutions (PAPD).
